# The first case report: diagnosis and management of necrotizing fusobacterium lung abscess via BALF next-generation sequencing

**DOI:** 10.1186/s12879-024-09087-5

**Published:** 2024-02-19

**Authors:** Yang Liu, Ziye Li, Handan Fu, Weiliang Ruan, Hua Wang, Yuhong Ding, Miao Zhang

**Affiliations:** 1 Respiratory Department, Shaoxing City Keqiao District Hospital of Traditional Chinese Medicine, 312030 Shaoxing, China; 2 Special Inspection Department, Shaoxing City Keqiao District Hospital of Traditional Chinese Medicine, 312030 Shaoxing, China; 3 Radiology Department, Shaoxing City Keqiao District Hospital of Traditional Chinese Medicine, 312030 Shaoxing, China

**Keywords:** Fusobacterium necrophorum, Lung abscess, Bronchoalveolar lavage fluid, Next-generation sequencing, Targeted antibiotic therapy

## Abstract

**Background:**

*Fusobacterium necrophorum (F. necrophorum)*-induced necrotizing pneumonia is a rare but severe pulmonary infection. Insufficient microbiological detection methods can lead to diagnostic difficulties.

**Methods:**

We report a case of *F. necrophorum* lung abscess diagnosed by next-generation sequencing (NGS) of bronchoalveolar lavage fluid (BALF).

**Results:**

BALF-NGS detected *F. necrophorum*, guiding subsequent targeted antibiotic therapy. With active drainage and metronidazole treatment, the patient’s condition was effectively treated.

**Conclusion:**

BALF-NGS is a valuable tool for the rapid diagnosis of infections caused by difficult-to-culture bacteria. It played a decisive role in the early identification of *F. necrophorum*, enabling timely and targeted antibiotic intervention. Early diagnosis and appropriate treatment are crucial for the management of *F. necrophorum* pneumonia.

## Introduction

*Fusobacterium necrophorum* is a Gram-negative, anaerobic bacterium that primarily colonizes the human oropharynx [1, 2]. In most cases, it plays an opportunistic pathogenic role, but can cause serious systemic infections [1], including Lemierre’s syndrome [3–4], when immunity is impaired or mucosal barriers are damaged. One study showed the incidence of *F. necrophorum* in adolescents with pharyngitis was 3.3% [5]. In recent years, advances in molecular techniques have greatly expanded our understanding of the *F. necrophorum* infection spectrum. In addition to traditional neck infections and sepsis, it can now cause lung abscesses, brain abscesses, pericarditis, liver abscesses, and ovarian abscesses [6–9], though relevant reports remain limited. We report a case of *F. necrophorum* lung abscess diagnosed using BALF-NGS to enhance understanding of this disease.

## Case report

**Fig. 1:**
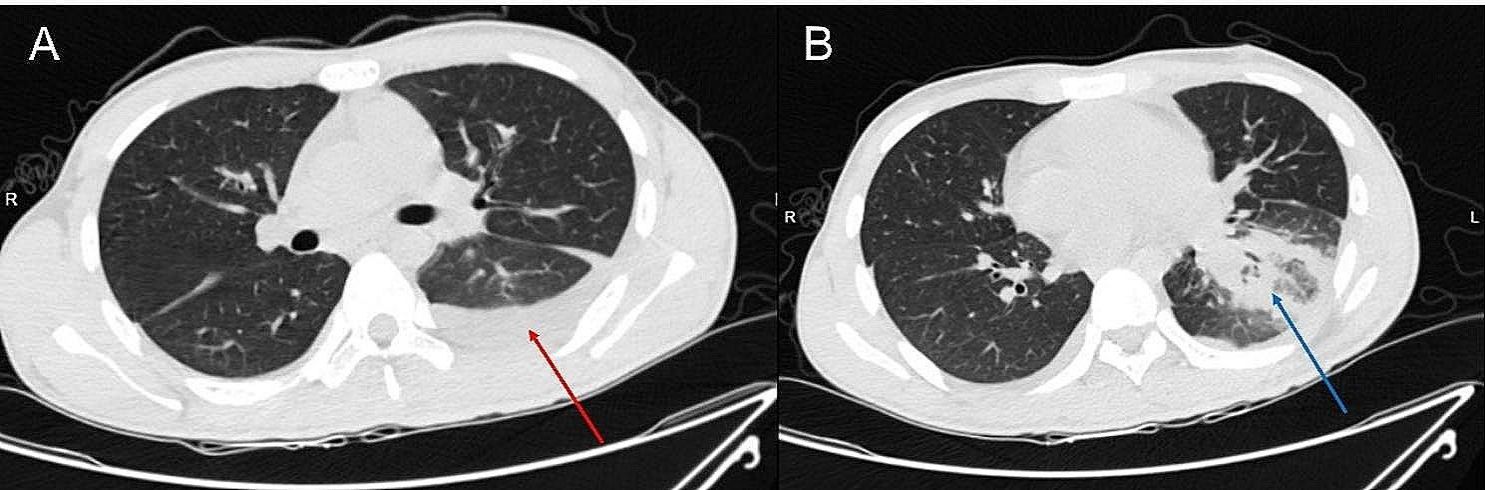
Chest CT images from day one of admission. (**A**) There is a small amount of pleural effusion on the left side, indicated by the red arrow. (**B**) Partial consolidation in the left lower lobe is highlighted with a blue arrow, suggestive of an infectious process within the lung parenchyma

**Fig. 2:**
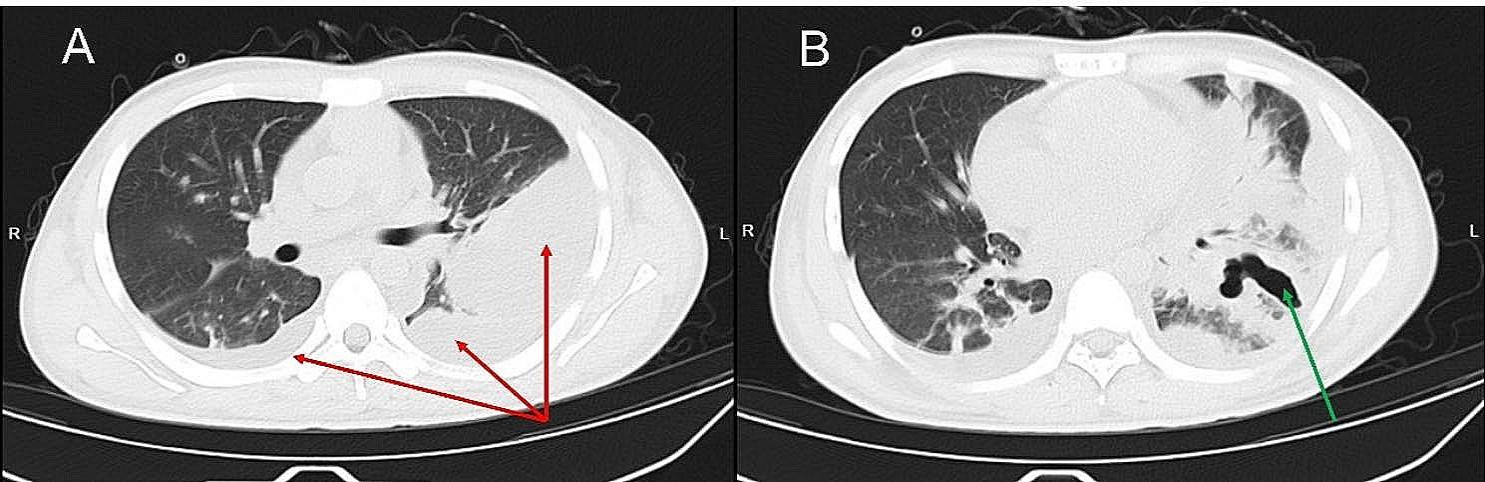
Chest CT images from day three of admission. (**A**) Increased pleural effusion in the left thoracic cavity, some of which is loculated, and a small effusion in the right thoracic cavity, both marked with red arrows. (**B**) Extensive consolidation in the left lower lung with cavity formation is denoted by a green arrow, indicating the development of a lung abscess

**Fig. 3:**
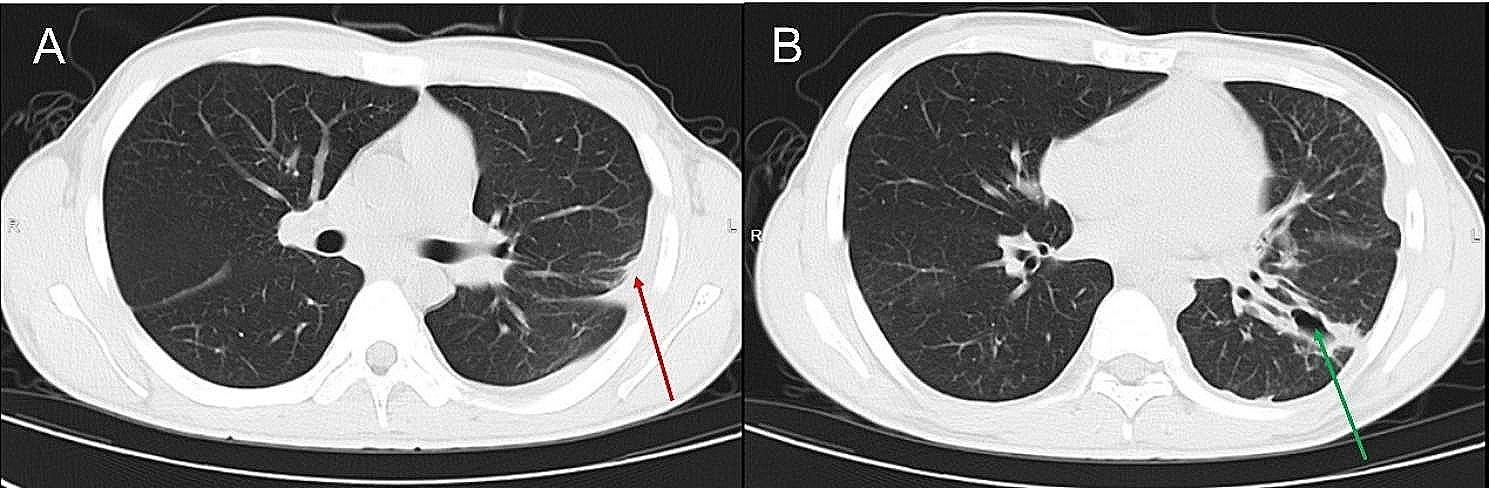
Chest CT images from day thirty of admission. (**A**) Compared to previous imaging, the right-sided pleural effusion has resolved, and there is a slight decrease in the loculated left pleural effusion, as shown by the red arrow. (**B**) The consolidation in the left lower lung has reduced, and the size of the cavity has decreased, as noted by the green arrow, reflecting a positive response to therapy

A 19-year-old male presented with a 10-day history of sore throat, 2 days of continuous chest pain, and 1 day of fever and respiratory distress, and was admitted on September 14, 2023. He had no previous medical history and a healthy lifestyle. Ten days before admission he developed sustained sore throat without obvious cause and took oral amoxicillin for about 5 days with no significant relief. Two days ago left chest pain developed which progressively worsened, and 1 day ago he developed chills, fever and respiratory difficulty. His peak temperature reached 39 °C. Chest CT on admission showed left lung infection foci and left pleural effusion (Fig. 1). Vital signs were: temperature 39.1 °C, pulse 122 beats/min, respiration 25 breaths/min, blood pressure 81/54 mmHg. Moist rales were auscultated over the left lung. Laboratory tests showed: white blood cell count 39.8 × 10^9^/L (cut-off: 3.5 × 10^9^/L∼9.5 × 10^9^/L), neutrophil count 37.7 × 10^9^/L, neutrophil ratio 94.8%; C-reactive protein (CRP) 310.4 mg/L (cut-off: 0∼4 mg/mL); Procalcitonin 48.8ng/ml (cut-off: 0∼0.5ng/mL). Blood culture and throat swab culture were performed initially. Additional testing included BALF culture and pleural drainage culture as part of the evaluation, but all culture results were negative. He was initially diagnosed with community-acquired pneumonia and empirically treated with ceftriaxone/sulbactam 2.0 g intravenous every 8 h combined with levofloxacin 0.5 g daily. Symptoms did not improve after 3 days of treatment. Repeat chest CT showed significant expansion of the lung infection range accompanied by cavity formation and encasing pleural effusion (Fig. 2). Bronchoscopy showed purulent secretions. BALF-NGS detected *F. necrophorum* with 35,030 reads. Based on medical history, clinical presentation and auxiliary examinations, he was definitively diagnosed with *F. necrophorum* lung abscess with pleural effusion. Treatment was adjusted to ceftriaxone/sulbactam 2.0 g intravenous every 8 h combined with metronidazole 0.5 g intravenous every 8 h for anti-infection, with chest tube drainage. Head CT, neck vascular ultrasound, liver, spleen and kidney ultrasound showed no abscesses in other organs or neck vein thrombosis, ruling out Lemierre’s syndrome. With treatment, symptoms and chest CT significantly improved (Fig. 3), and he was discharged after 31 days with recovery.

## Discussion

*F. necrophorum* is a rare disease, with an incidence of approximately 2.8 cases per million people in Denmark [10] and around 19 cases per year in England and Wales, mostly affecting those aged 16–23 years [6]. This case report describes a 19-year-old male patient diagnosed with *F. necrophorum* lung abscess. Notably, this is the first reported case diagnosing this disease using BALF-NGS, highlighting the value of this technique. *Fusobacterium* is a normal commensal residing in the human oral cavity, upper gastrointestinal tract, intestines and urogenital tract [1, 2], acting as an opportunistic pathogen in various necrotizing infections following skin and mucosal damage, causing pharyngeal and gingival abscesses, otitis media, sinusitis and more [11]. It can cause multi-organ abscess formation during invasive infections [6–9]. Our patient was a 19-year-old male with a 10-day history of sore throat as an important clue for *Fusobacterium* infection. *Fusobacterium* can spread from the oropharynx to lung tissue via inhalation or hematogenous route, proliferating in the alveoli to cause pulmonary necrosis and abscess formation [12].

*Fusobacterium* is difficult to detect by conventional bacterial culture methods [13], often leading to missed treatment opportunities when culture results are negative. The cultures of blood, BALF, pleural drainage and throat swab samples in this case were all negative for bacterial growth. This demonstrated the limitations of standard culture techniques for detecting *F. necrophorum*. In contrast, BALF-NGS was able to rapidly detect *F. necrophorum* and establish the diagnosis, highlighting the advantage of this technique when cultures fail. PCR is also an important tool for *Fusobacterium* detection [14, 15], but its application is limited. In recent years, high-throughput gene sequencing (NGS) has been widely applied in infectious disease diagnosis. It has the advantage of directly detecting pathogen genetic material from clinical samples without relying on bacterial culture [16, 17]. Application of BALF-NGS has significantly improved detection of causative bacteria in pulmonary infections. Studies show that compared to conventional culture, BALF-NGS can increase bacterial detection rates in pneumonia patients by up to 25% [18], especially for diagnosis of rare pathogenic bacteria and lung infections in immunocompromised hosts [19, 20]. Cases of *Fusobacterium* infection diagnosed by NGS have been reported [21]. However, to our knowledge there are no reports using BALF-NGS to detect *F. necrophorum* lung abscess previously. This case provides important reference value as the first report using BALF-NGS for the diagnosis of *F. necrophorum* lung abscess.

The choice of a sensitive antibiotic according to susceptibility is critical for *Fusobacterium* infection treatment. Penicillin, metronidazole, clindamycin and chloramphenicol have good activity against it [22]. Metronidazole is the first-line drug for *Fusobacterium* infections [23]. In this case, significant clinical improvement occurred after adjusting treatment to metronidazole. Active drainage of abscesses is also extremely important alongside antibiotic therapy, with some cases potentially requiring intrapleural fibrinolysis injection [24] or thoracoscopic surgery [25]. Despite the challenges of obtaining positive culture results, as seen in this case with multiple negative culture outcomes, the endeavor to secure a culture for antimicrobial susceptibility testing cannot be overstated. This is crucial not only for confirming the pathogen but also for tailoring the antibiotic treatment to the specific susceptibilities of the organism, which is a key step in the management of infections such as those caused by *Fusobacterium*. Consequently, while we highlight the diagnostic prowess of BALF-NGS, we also recognize the irreplaceable role of traditional culture methods in guiding the most effective therapeutic interventions.

Mortality rates specifically for *F. necrophorum* lung abscess have not yet been reported. However, the 30-day mortality for *Fusobacterium* bacteremia is 21.1% [26], with age and comorbidities associated with higher risk of death [27]. Given our patient’s rapid progression despite initial combination broad-spectrum antibiotics, prognosis would likely have been poor without early targeted treatment. In general, mortality depends on factors including pathogen virulence, extent of involvement, and timeliness and appropriateness of treatment.

However, there are some limitations. As a single case report, conclusions have limited generalizability. Detailed mechanisms of how *Fusobacterium* causes necrotizing pneumonia and abscesses remain unclear. Further large cohort studies and basic research are still needed.

In summary, this case identified *F. necrophorum* using BALF-NGS, guiding adjustment of successful treatment. This technique has important significance for accurate diagnosis of similar cases. The report also highlights the critical role of targeted antibiotic therapy in managing lung abscesses.

## Data Availability

All data generated or analyzed during this study are included in this published article. For any requests of the data, please contact the corresponding author Yang Liu.
